# Long-term trends in the burden of non-rheumatic calcific aortic valve disease in China from 1990 to 2021, with projections to 2050: results from the global burden of disease study

**DOI:** 10.3389/fmed.2025.1690631

**Published:** 2025-10-29

**Authors:** Bin Jian, Zhen Li, Yu Huang, Zhixiong Wei, Gaoyang Zhao, Jun Fang

**Affiliations:** ^1^Department of Cardiology, Fujian Medical University Union Hospital, Fujian Cardiovascular Medical Center, Fujian Institute of Coronary Artery Disease, Fujian Cardiovascular Research Center, Fujian Medical University Heart Center, Fuzhou, China; ^2^Department of Cardiology, Renmin Hospital, Hubei University of Medicine, Shiyan, China; ^3^Department of Cardiology, Xinyang Central Hospital, Xinyang, China

**Keywords:** non-rheumatic calcific aortic valve disease, incidence, prevalence, mortality, disability-adjusted life years, global burden of disease, Bayesian age-period-cohort model

## Abstract

**Background:**

Non-rheumatic calcific aortic valve disease (NRCAVD) has emerged as a significant health challenge globally, with China’s rapidly aging population bearing a disproportionate burden, necessitating comprehensive epidemiological analysis to guide public health strategies.

**Methods:**

Utilizing data from the Global Burden of Disease (GBD) database 2021, we examined the incidence, prevalence, mortality, and disability-adjusted life years (DALYs), along with their corresponding age-standardized rates (ASRs), of NRCAVD in China from 1990 to 2021 and compared these data with global trends. The analysis employed joinpoint regression to calculate the estimated annual percentage change (EAPC) and average annual percentage change (AAPC) for evaluating NRCAVD trends over the past 32 years. Additionally, we employed the Bayesian age–period–cohort model to project these trends through 2050.

**Results:**

The age-standardized incidence and prevalence rates of NRCAVD in China increased from 1.42 to 2.52 per 100,000 and 17.24 to 32.68 per 100,000, respectively, between 1990 and 2021. Favorably, the age-standardized mortality and disability-adjusted life year rates of NRCAVD decreased from 0.10 to 0.07 per 100,000 and 2.42 to 1.92 per 100,000, respectively. The rates were higher in male individuals than in female individuals across all four metrics over the study period. In China, the estimated annual percentage changes for incidence, prevalence, mortality, and disability-adjusted life years were 2.04, 2.33, −1.34%, and −1.06%, respectively, while the average annual percentage changes for incidence, prevalence, mortality, and disability-adjusted life years were 0.04, 0.50, 0.00%, and −0.02%, respectively. The projections from Bayesian age–period–cohort model showed that the burden of NRCAVD is expected to continue through 2050.

**Conclusion:**

The burden of NRCAVD in China has dramatically increased from 1990 to 2021, with a notable rise associated with aging. Male individuals appear to be more susceptible than female individuals and face higher mortality risks associated with NRCAVD in China. The forecast suggests that this trend will persist until 2050, highlighting NRCAVD as a significant public health challenge in China over the next three decades.

## Highlights

- This study comprehensively assesses China’s NRCAVD disease burden (1990–2050).
- Male individuals show higher susceptibility and mortality risk than female individuals in China.
- NRCAVD poses a growing public health threat in China, driven by its demographic profile.

## 1 Introduction

With economic development and healthcare improvements, the incidence of new cases of rheumatic heart disease appears to be decreasing (1–4). In contrast, the number of new cases of non-rheumatic valvular heart disease, which include calcific aortic valve disease and degenerative mitral valve disease, among others, has been gradually increasing, largely due to the aging population (5–7). Non-rheumatic calcific aortic valve disease (NRCAVD), one of the most important subtypes of non-rheumatic valvular heart diseases, imposes a heavy burden both in China and globally, posing an increasing public health threat to older adults. Fortunately, NRCAVD can be treated, especially since the groundbreaking transcatheter aortic valve replacement (TAVR) procedure was successfully performed for the first time in 2002. This therapeutic method has been a major milestone over the last 20 years (8), attracting increasing attention from researchers focusing on the disease.

A previous study reported that there were more than 9.4 million cases of NRCAVD and 127,000 deaths globally in 2019, contributing to a heavy burden and posing an increasing threat to public health due to the aging global population (9). As the country with the largest aging population, China has experienced an explosive increase in new NRCAVD cases over the years. Despite the latest updates from the GBD database in 2021, there have been no studies estimating the burden of NRCAVD in China. Therefore, based on previous studies and the most updated GBD data, we conducted a comprehensive analysis of the incidence, prevalence, mortality, and disability-adjusted life years (DALYs) of NRCAVD in China and compared this burden with global trends from 1990 to 2021. Meanwhile, we used a Bayesian age–period–cohort projection model to forecast the trends of NRCAVD in China over the next three decades until 2050, based on demographic changes and historical data.

## 2 Methods

### 2.1 Data source

In this study, data related to NRCAVD (ICD-10 code: I35.0-I35.9) were obtained from the Global Burden of Disease (GBD) database (2021), and the associated tools were used. To quantify temporal patterns and assess trends in incidence, prevalence, mortality, and DALYs, along with their corresponding age-standardized rates (ASRs)—including age-standardized incidence rate (ASIR), age-standardized prevalence rate (ASPR), age-standardized mortality rate (ASMR), and age-standardized DALYs rate (ASDALYs)—of NRCAVD from 1990 to 2021, we also calculated the estimated annual percentage changes (EAPCs) and average annual percentage changes (AAPCs) over the study period. Using GBD tools, we analyzed scatterplot data from the GBD database^1^ for China and globally from 1990 to 2021, and we used a Bayesian age-period-cohort model to project future trends in the NRCAVD burden in China until 2050.

### 2.2 Statistical analysis

The incidence, prevalence, mortality, and DALY cases, along with their corresponding rates (ASIR, ASPR, ASMR, ASDALYs), were the primary indicators used to describe the burden of NRCAVD. Each rate was reported per 100,000 population and accompanied by a 95% uncertainty interval (95% UI), which was calculated using the GBD algorithm. The dynamics of NRCAVD were analyzed by calculating EAPCs and AAPCs to identify temporal trends in the disease burden; the 95% CIs for EAPCs and AAPCs were determined using linear modeling. Accordingly, the rates were modeled using the equation: *y* = *α* + *β*x + *ε*, where *y* = ln (ASR) and *x* = calendar year. The EAPC was calculated as: 100 × (exp (β) -1), and the AAPC was calculated as (10):


$$\;\mathrm{AA}PC=\{e\mathrm{xp}(\frac{\sum w_{i}b_{i}}{\sum w_{i}})\}\times 100$$

where *b* is the slope coefficient for the *I^th^* segment, with *i* indexing the segments within the desired range of years, and *w_i_* is the length of each segment in years.

If the upper limit of the EAPC or AAPC and its 95% CI was negative, its corresponding rate showed a decreasing trend. On the contrary, if the lower limit of the EAPC or AAPC and its 95% CI was positive, its corresponding rate showed an increasing trend. If it included 0, it indicated a stable trend. Joinpoint regression analysis was employed to identify significant temporal change points, known as joinpoints, where the slope of the trend experiences a statistically significant shift. The model can be expressed mathematically as (11):


$$\log(Y_{t})=\beta_{0}+\beta_{1}t+\sum_{j=1}^{k}\;\beta_{j+1}\;{\left(t-\tau_{j}\right)}_{+}$$

where *Y*_*t*_ represents the observed response variable at time *t*; *β*_0_ denotes the baseline intercept, *β*_1_ represents the initial slope, *τj* indicates the *j*-th joinpoint location, and *β*_*j* + 1_ quantifies the slope change after *τj*, with (*t* − *τj*) + implementing the piecewise linear structure.

We used a Bayesian age–period–cohort model incorporating integrated nested Laplace approximations to project future trends of the NRCAVD burden. The model used the following formula (12):


$$\log(Y_{\left\{a,p+t\right\}})=\mu +\alpha_{a}+\beta_{\left\{p+t\right\}}+\gamma_{\left\{c+t\right\}}+\delta_{\left\{a,p+t\right\}}$$

where *Y* is the forecast year, *p* is the reference year, *t* is the forecast time step, *μ* is the global intercept, *α* is the age effect, *β* is the period effect, *γ* is the cohort effect, and *δ* is the interaction term.

The global base map was obtained from the Natural Earth repository^2^, a publicly accessible and politically neutral geographic data source. The processing and visualization of geospatial data were performed programmatically using the rnaturalearth package in R, with full documentation available at https://cran.r-project.org/web/packages/rnaturalearth/vignettes/rnaturalearth.html. All calculations were performed using R Studio (version 4.1.3) (R Project for Statistical Computing) and the Joinpoint software program (version 4.9.1.0). All *p*-values were two-tailed, and a *p*-value of < 0.05 was considered statistically significant.

## 3 Results

### 3.1 The burden of NRCAVD in China and worldwide

#### 3.1.1 Incidence of NRCAVD in China and worldwide

**Figure 1:**
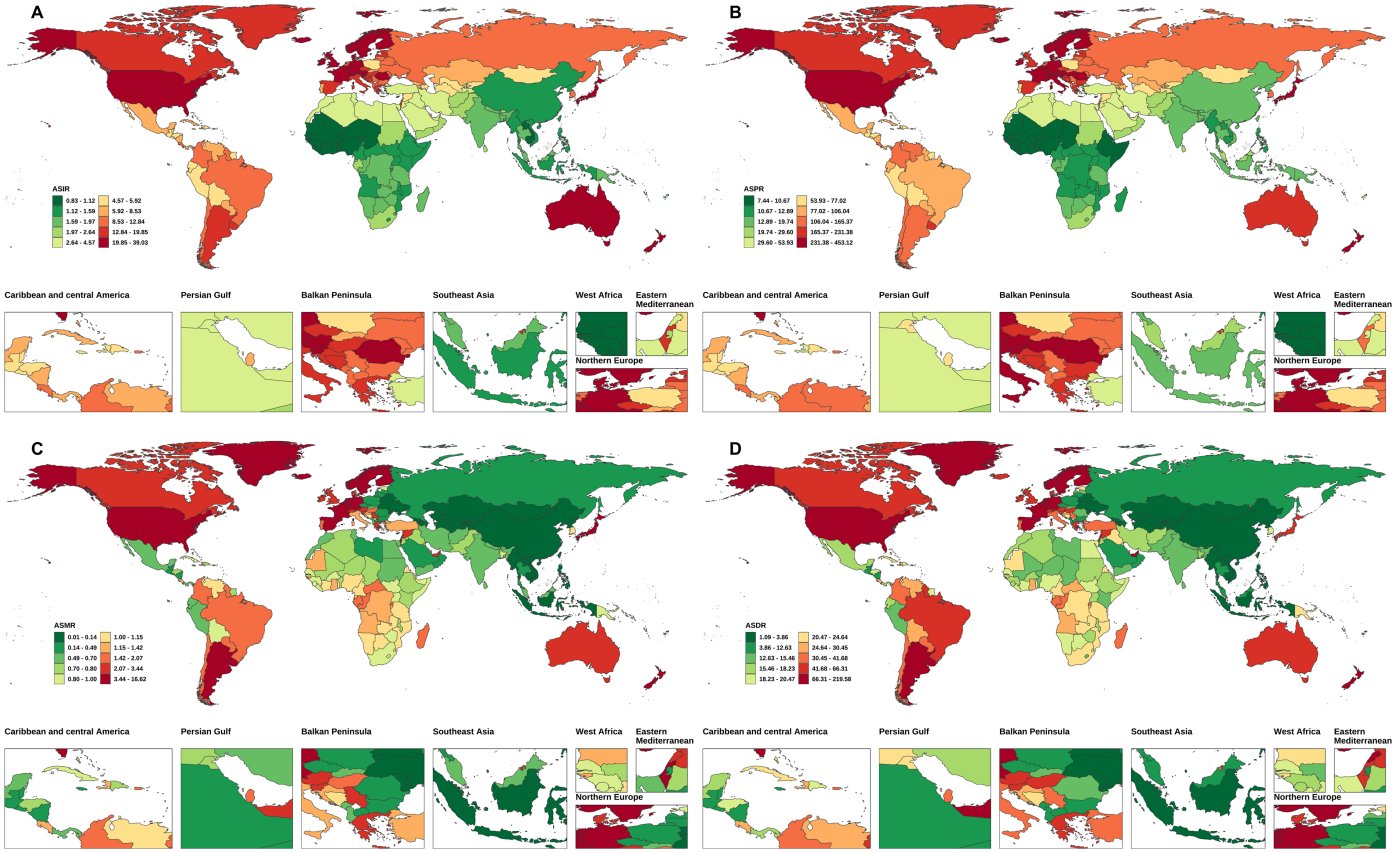
The ASIR, ASPR, ASMR, and ASDALYs of NRCAVD in China and globally in 1990. **(A)** ASIR, **(B)** ASPR, **(C)** ASMR, **(D)** ASDALYs. ASIR, age-standardized incidence rate; ASPR, age-standardized prevalence rate; ASMR, age-standardized mortality rate; ASDALYs, age-standardized DALYs rate.

**Figure 2:**
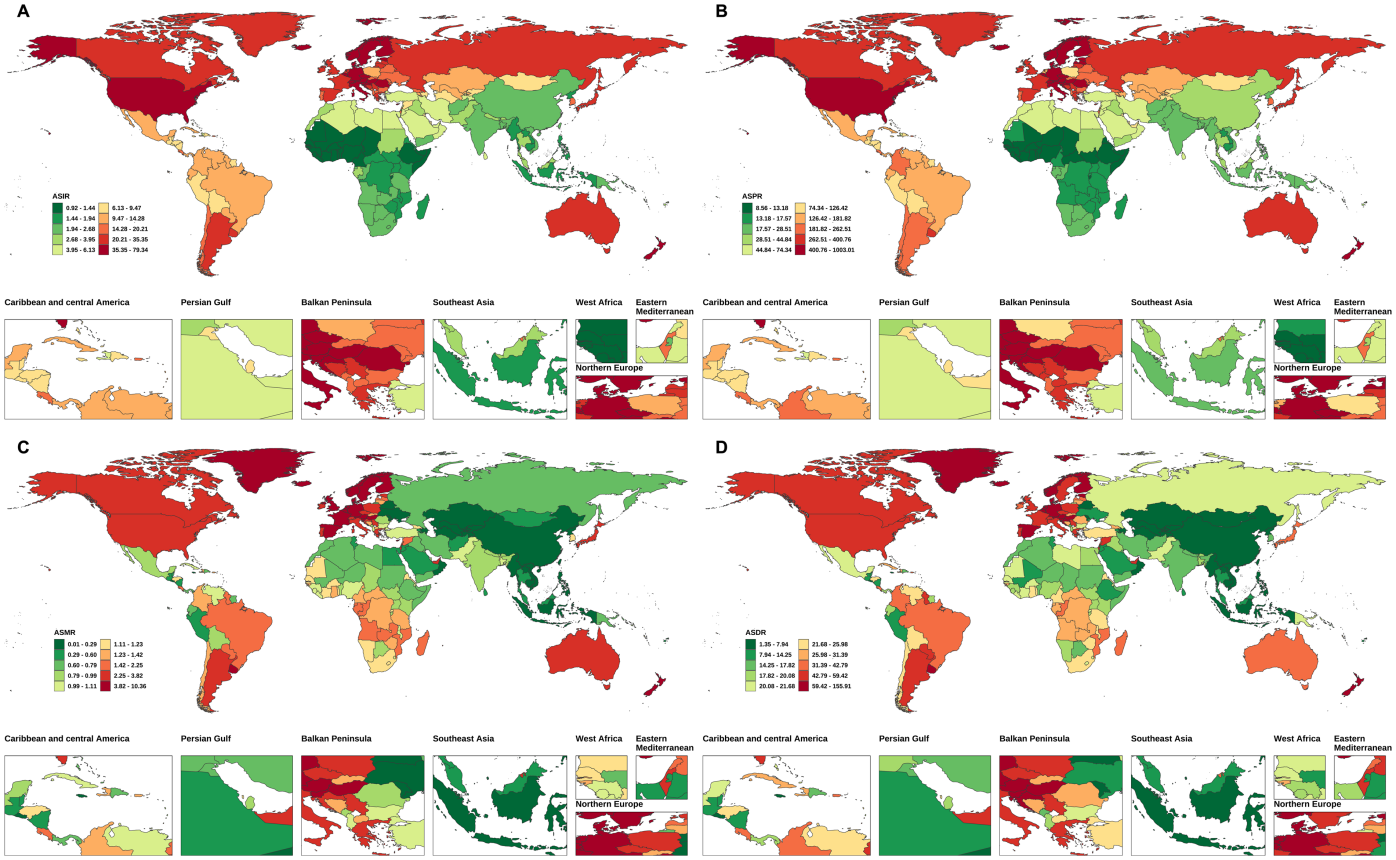
The ASIR, ASPR, ASMR, and ASDALYs of NRCAVD in China and globally in 2021. **(A)** ASIR, **(B)** ASPR, **(C)** ASMR, **(D)** ASDALYs. ASIR, age-standardized incidence rate; ASPR, age-standardized prevalence rate; ASMR, age-standardized mortality rate; ASDALYs, age-standardized DALYs rate.

The number of NRCAVD cases in China increased from 12,038 (95% UI: 9,320–14,911) in 1990 to 54,642 (95% UI: 42,318–66,940) in 2021, representing a cumulative increase of 353.91%. Meanwhile, globally, the incidence increased from 396,981 cases (95% UI: 329,260–464,090) in 1990 to 1,044,370 cases (95% UI: 906,615–1,179,672) in 2021, representing a cumulative increase of 163.08%. In China, the ASIR increased from 1.42 (95% UI: 1.09–1.72) per 100,000 population in 1990 to 2.52 (95% UI: 1.95–3.06) per 100,000 population during the same period. Globally, the ASIR rose from 10.17 (95% UI: 8.48–11.86) per 100,000 population in 1990 to 12.03 (95% UI: 10.43–13.56) per 100,000 population in 2021(Table 1 and Figures 1A, 2A). From 1990 to 2021, the EAPC and AAPC in the incidence rate in China increased by 2.04% (95% CI: 1.96–2.11) and 0.04% (95% CI: 0.04–0.04), respectively. Globally, the EAPC and AAPC in the incidence rate increased by 0.79% (95% CI: 0.70–0.89) and 0.06% (95% CI: 0.06–0.06), respectively (Tables 1, 2).

**Table 1:** All-age cases and ASRs of the incidence, prevalence, mortality, and DALYs, along with their corresponding EAPCs, of NRCAVD in China and globally in 1990 and 2021.

Area	1990 (95% UI)		2021 (95% UI)		1990–2021 (95% CI)
	All-ages cases	ASRs per 100,000	All-ages cases	ASRs per 100,000	EAPCs
China					
Incidence	12,038 (9320–14,911)	1.42 (1.09–1.72)	54,642 (42318–66,940)	2.52 (1.95–3.06)	2.04 (1.96–2.11)
Prevalence	130,941 (98721–164,981)	17.24 (13.08–21.54)	685,550 (527566–848,322)	32.68 (25.06–40.23)	2.33 (2.22–2.44)
Mortality	654 (399–1,002)	0.10 (0.06–0.15)	1,335 (1031–1786)	0.07 (0.06–0.10)	−1.34 (−1.57--1.11)
DALYs	20,767 (12942–30,740)	2.42 (1.57–3.54)	36,639 (28315–47,287)	1.92 (1.49–2.48)	−1.06 (−1.22--0.89)
Global					
Incidence	396,981 (329260–464,090)	10.17 (8.48–11.86)	1,044,370 (906615–1,179,672)	12.03 (10.43–13.56)	0.79 (0.70–0.89)
Prevalence	4,686,910 (3874992–5,539,483)	129.75 (106.01–152.98)	13,320,896 (11422539–15,249,411)	158.35 (135.92–181.00)	0.88 (0.77–0.99)
Mortality	57,932 (52886–61,950)	1.92 (1.73–2.07)	142,205 (120675–155,575)	1.83 (1.54–2.00)	−0.05 (−0.16–0.06)
DALYs	1,121,945 (1040350–1,218,803)	32.00 (29.43–34.67)	2,243,000 (2004168–2,459,248)	27.74 (24.67–30.48)	−0.39 (−0.49--0.28)

ASRs, age-standardized rates; EAPCs, estimated annual percentage changes; DALYs, disability-adjusted life years.

**Table 2:** The AAPC in the ASIR, ASPR, ASMR, and ASDALYs of NRCAVD in China and Globally from 1990 to 2021.

Area	**ASIR**			**ASPR**			**ASMR**			**ASDALYs**		
	**Period**	**APC**	**AAPC**	**Period**	**APC**	**AAPC**	**Period**	**APC**	**AAPC**	**Period**	**APC**	**AAPC**
China	1990–2003	1.53 (1.48–1.58) *	0.04 (0.04–0.04)	1990–2004	1.69 (1.64–1.74) *	0.50 (0.49–0.51)	1990–1998	−0.22 (−0.29--0.16)	0 (−0.00–0.00)	1990–2004	−0.06 (−0.12–0.01)	−0.02 (−0.02--0.02)
	2003–2010	2.83 (2.63–3.03) *		2004–2009	3.78 (3.43–4.13) *		1998–2004	0.59 (0.34–0.84) *		2004–2006	−3.35 (−4.43--2.25) *	
	2010–2019	1.92 (1.82–2.02) *		2009–2019	2.11 (2.02–2.20) *		2004–2013	−3.54 (−3.74--3.35) *		2006–2014	−2.41 (−2.55--2.26) *	
	2019–2021	0.35 (−0.68–1.40)		2019–2021	−0.06 (−1.73–1.63)		2013–2021	0.17 (−0.02–0.36) *		2014–2021	0.45 (0.30–0.61) *	
Global	1990–2000	0.31 (0.24–0.37) *	0.06 (0.06–0.06)	1990–2000	0.65 (0.60–0.71) *	0.94 (0.92,0.96)	1990–1993	0.22 (−0.19–0.62) *	0 (−0.00–0.00)	1990–1993	−0.91 (−1.11--0.72) *	−0.14 (−0.15- -0.13)
	2000–2010	1.56 (1.50–1.62) *		2000–2010	1.60 (1.55–1.65) *		1993–2004	−0.51 (−0.90--0.11) *		1993–2003	0.09 (0.08–0.10) *	
	2010–2016	0.25 (0.14–0.36) *		2010–2016	0.23 (0.12–0.33) *		2004–2015	0.86 (0.78–0.94) *		2003–2015	0.43 (0.35–0.51) *	
	2016–2021	−0.41 (−0.52--0.30) *		2016–2021	−0.61 (−0.72--0.50)*		2015-2021	−1.34 (−1.65--1.04) *		2015–2021	−1.26 (−1.48--1.04)	

* means *p*-values < 0.05 and significant results; ASIR, age-standardized incidence rate; ASPR, age-standardized prevalence rate; ASMR, age-standardized mortality rate; ASDALYs, age-standardized DALYs rate; APC, annual percentage change; AAPC, average annual percentage change.

#### 3.1.2 Prevalence of NRCAVD in China and worldwide

Regarding prevalence, the cases of NRCAVD in China increased from 130,941 (95% UI: 98,721–164,981) in 1990 to 685,550 (95% UI: 527,566–848,322) in 2021, representing a cumulative increase of 423.56%. Simultaneously, globally, the prevalence increased from 4,686,910 (95% UI: 3,874,992–5,539,483) in 1990 to 13,320,896 (95% UI: 11,422,539–15,249,411) in 2021, representing a cumulative increase of 184.21%. Globally, the ASPR increased from 129.75 (95% UI: 106.01–152.98) per 100,000 population in 1990 to 158.35 (95% UI: 135.92–181.00) per 100,000 population in 2021. In China, the ASPR increased from 17.24 (95% UI: 13.08–21.54) per 100,000 population in 1990 to 32.68 (95% UI: 25.06–40.23) per 100,000 population in 2021 (Table 1 and Figures 1B, 2B). From 1990 to 2021, the global EAPC and AAPC in the prevalence increased by 0.88% (95% CI: 0.77–0.99) and 0.94% (95% CI: 0.92–0.96), respectively, while in China, it increased by 2.33% (95% CI: 2.22–2.44) and 0.50% (95% CI: 0.49–0.51), respectively (Tables 1, 2).

#### 3.1.3 Mortality rate of NRCAVD in China and worldwide

Globally, NRCAVD caused 57,932 (95% UI: 52886–61,950) deaths in 1990, which increased to 142,205 (95% UI: 120,675–155,575) in 2021, representing a 145.47% increase compared to 1990. Meanwhile, in China, the number of deaths increased from 654 (95% UI: 399–1,002) in 1990 to 1,335 (95% UI: 1031–1786), and the mortality rate increased by 104.13% from 1990 to 2021. The global ASMR decreased from 1.92 (95% UI: 1.73–2.07) per 100,000 population in 1990 to 1.83 (95% UI: 1.54–2.00) per 100,000 population in 2021. In China, the ASMR decreased from 0.10 (95% UI: 0.06–0.15) per 100,000 population in 1990 to 0.07 (0.06–0.10) per 100,000 population in 2021 (Table 1 and Figures 1C, 2C). From 1990 to 2021, the global EAPC in the mortality rate decreased by −0.05% (95% CI: −0.16 – 0.06), while in China, it decreased by −1.34% (95% CI: −1.57 – –1.11). Nevertheless, the AAPC in the mortality rate from 1990 to 2021 was 0% (95% CI: −0.00 – 0.00) for both the global population and China (Table 2).

#### 3.1.4 DALYs of NRCAVD in China and worldwide

Globally, the number of DALYs due to NRCAVD was 1,121,945 (95% UI: 1,040,350–1,218,803) in 1990 and 2,243,000 (95% UI: 2,004,168–2,459,248) in 2021, representing a 99.92% increase compared to 1990 (Table 1). In China, the number of DALYs increased by 76.43% from 1990 to 2021 (Table 1). Globally, the ASDALYs decreased from 32.00 (95% UI: 29.43–34.67) per 100,000 population in 1990 to 27.74 (95% UI: 24.67–30.48) per 100,000 population in 2021. In China, the ASDALYs decreased from 2.42 (95% UI: 1.57–3.54) per 100,000 population in 1990 to 1.92 (95% UI: 1.49–2.48) per 100,000 population in 2021(Table 1 and Figures 1D, 2D). From 1990 to 2021, the global EAPC and AAPC in DALYs decreased by −0.39% (95% CI: −0.49 – –0.28) and −0.14% (95% CI: −0.15 – –0.13), respectively. In China, these values decreased by −1.06% (95% CI: −1.22 – –0.89) and −0.02% (95% CI: −0.02 – –0.02), respectively (Tables 1, 2).

### 3.2 Trends in the burden of NRCAVD in China and worldwide

**Figure 3:**
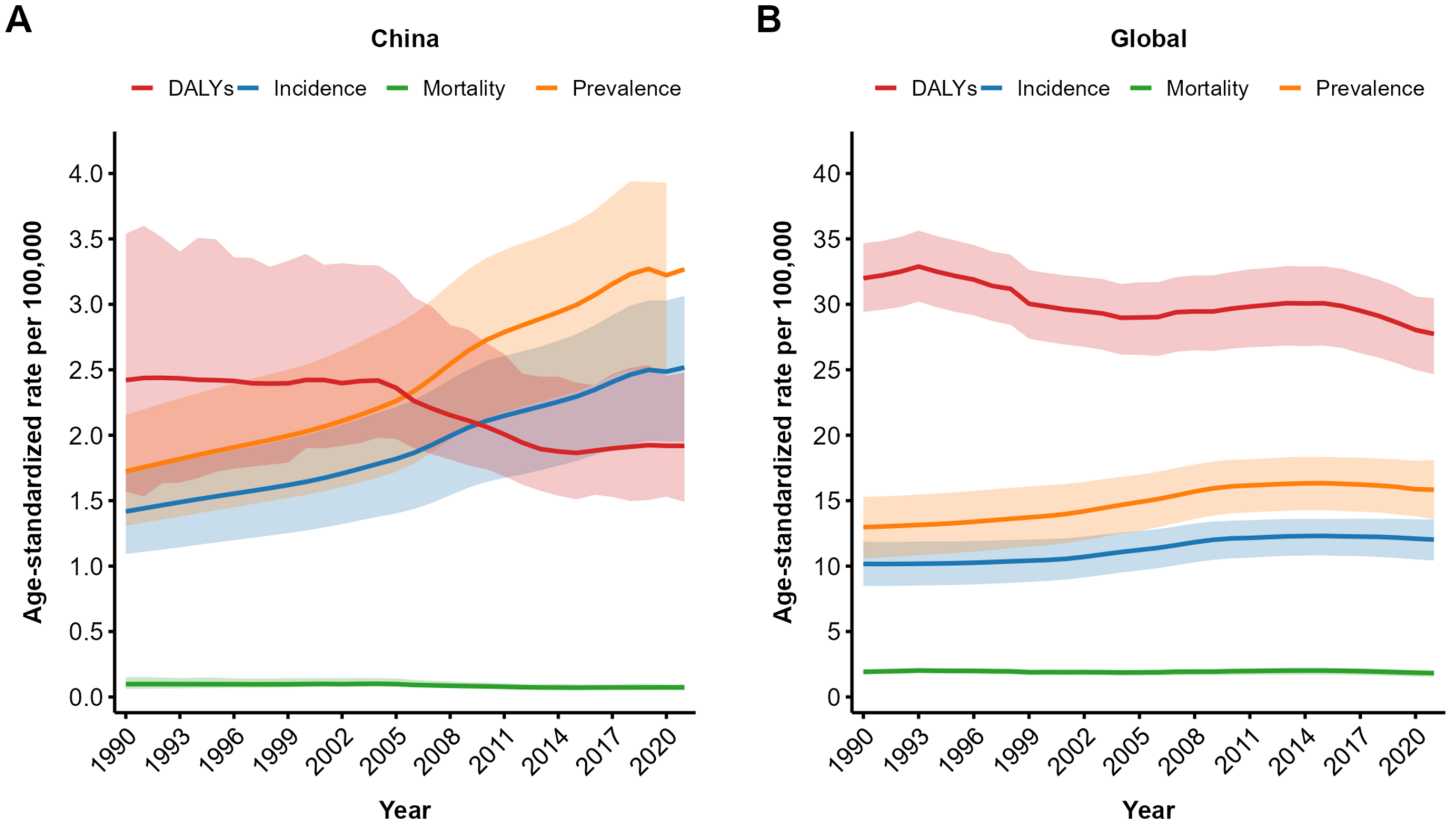
Trend comparison of the ASIR, ASPR, ASMR, and ASDALYs of NRCAVD in China and globally from 1990 to 2021. **(A)** Trends in China; **(B)** Global trends. ASIR, age-standardized incidence rate; ASPR, age-standardized prevalence rate; ASMR, age-standardized mortality rate; ASDALYs, age-standardized DALYs rate; DALYs, disability-adjusted life years. The shaded areas represent the 95% confidence intervals of the respective trend lines.

From 1990 to 2021, the ASIR of NRCAVD showed a consistent increase both in China and globally. In China, the ASPR exhibited a significant upward trend until 2019, followed by a transient decline and a slight rebound after 2020. Globally, the ASPR rose steadily until 2015, then gradually decreased from 2016 to 2021. While the ASMR in China demonstrated an overall decline, the global ASMR remained relatively stable throughout the period. The ASDALYs in China decreased notably between 2004 and 2014 but increased after 2014. Globally, the ASDALYs fluctuated through multiple phases of increase and decrease, culminating in a significant decline from 2015 to 2021 (Figure 3).

### 3.3 Joinpoint regression analysis of the burden of NRCAVD in China and worldwide

**Figure 4:**
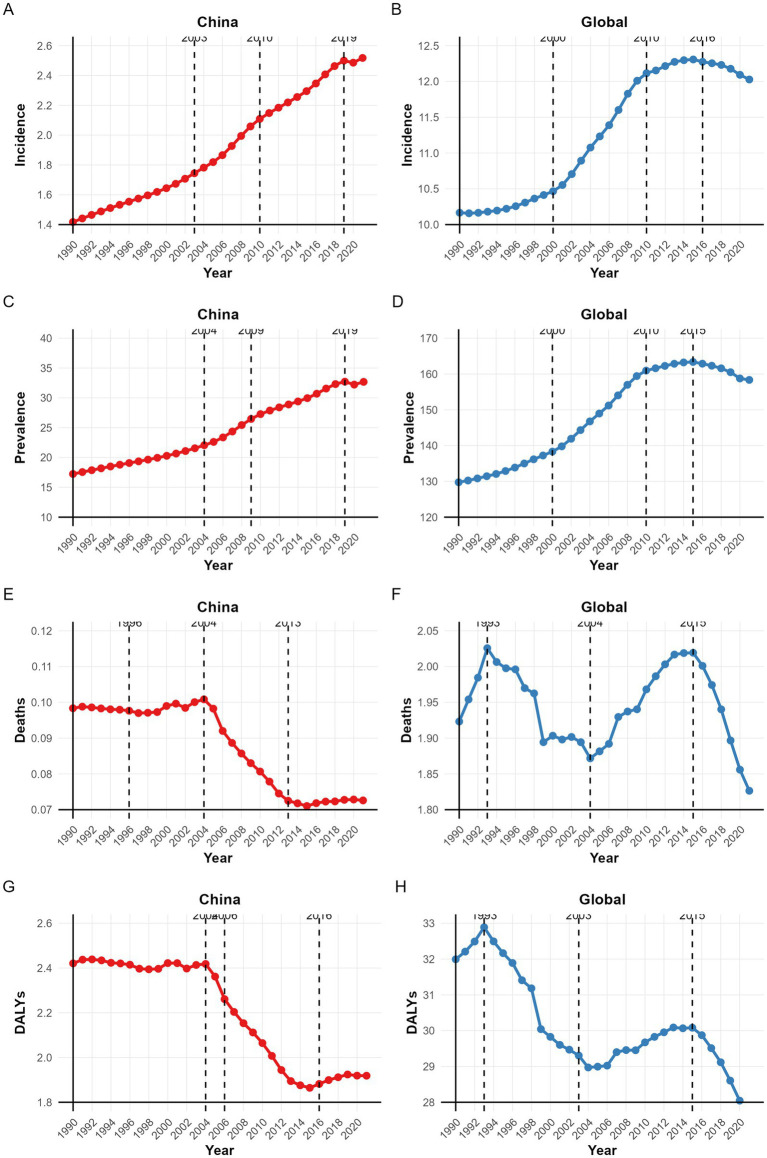
The APC in the ASIR, ASPR, ASMR, and ASDALYs of NRCAVD in China and globally from 1990 to 2021. **(A)** Incidence in China; **(B)** Global incidence; **(C)** Prevalence in China; **(D)** Global prevalence; **(E)** Mortality in China; **(F)** Global mortality; **(G)** DALYs in China; **(H)** Global DALYs. ASIR, age-standardized incidence rate; ASPR, age-standardized prevalence rate; ASMR, age-standardized mortality rate; ASDALYs, age-standardized DALYs rate; DALYs, disability-adjusted life years.

Joinpoint regression analysis revealed distinct temporal patterns in the ASIR, ASPR, ASMR, and ASDALYs of NRCAVD between 1990 and 2021 (Table 2 and Figure 4). In China, the ASIR and ASPR increased steadily until 2019 (both *p* < 0.05). After 2019, ASIR growth slowed (APC = 0.36, *p* > 0.05), while the ASPR slightly decreased (APC = −0.05, *p* > 0.05). The ASMR declined initially until 1998, rose between 1998 and 2004 (APC = 0.55, *p* < 0.05), decreased significantly from 2004 to 2013 (APC = −3.6, *p* < 0.05), and increased again from 2013 to 2021 (APC = 0.29, *p* < 0.05). The ASDALYs decreased consistently until 2014, with a pronounced reduction between 2004 and 2014 (APC = −2.98 to −2.42, *p* < 0.05), followed by an increase from 2014 to 2021 (APC = 0.52, *p* < 0.05) (Table 2 and Figure 4).

Globally, the ASIR and ASPR increased significantly from 1990 to 2016 (*p* < 0.05), then declined through 2021 (ASIR APC = −0.49; ASPR APC = −0.61; both *p* < 0.05). Both the ASMR and ASDALYs exhibited M-shaped trends: an initial rise from 1990 to 1993 (ASMR APC = 1.73; ASDALYs APC = 0.92; both *p* < 0.05), a subsequent decline (ASMR: 1993–2004, APC = −0.76; ASDALYs: 1993–2003, APC = −1.27; both *p* < 0.05), another increase (ASMR: 2004–2015, APC = 0.82; ASDALYs: 2003–2015, APC = 0.40; both *p* < 0.05), and a significant decrease from 2015 to 2021 (ASMR APC = −1.74; ASDALYs APC = −1.54; both *p* < 0.05) (Table 2 and Figure 4).

### 3.4 Sex disparities in the burden of NRCAVD across various age groups in China

**Figure 5:**
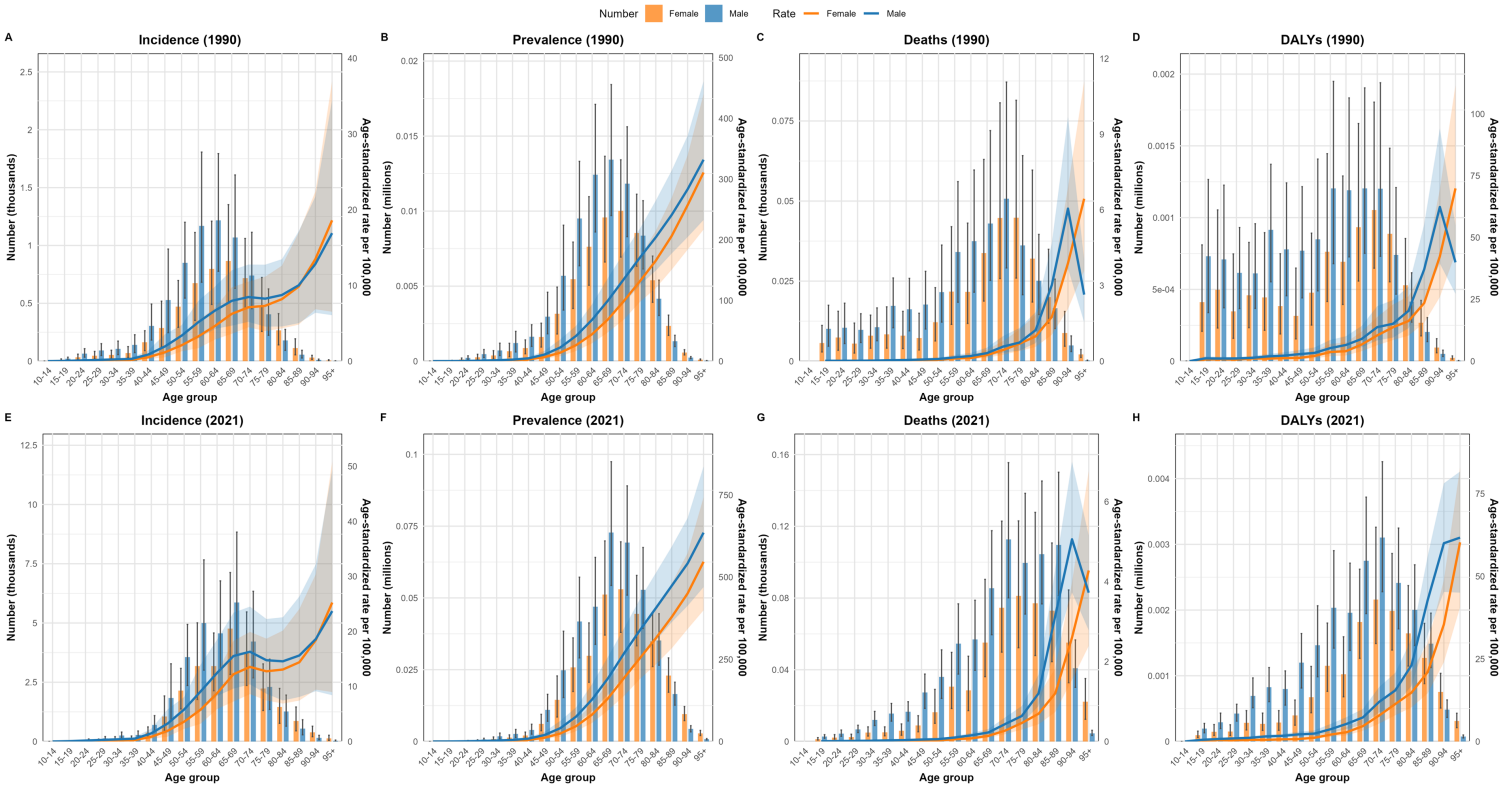
Comparison of the number of incidence, prevalence, mortality, and DALY cases in male and female individuals, along with their ASRs, across various age groups in China in 1990 and 2021. **(A)** Incidence in 1990; **(B)** Prevalence in 1990; **(C)** Deaths in 1990; **(D)** DALYs in 1990; **(E)** Incidence in 2021; **(F)** Prevalence in 2021; **(G)** Deaths in 2021; **(H)** DALYs in 2021. DALYs, disability-adjusted life years.

Figure 5 illustrates the incidence, prevalence, mortality, and DALYs of NRCAVD across various age groups in male and female individuals, along with their ASRs in China between 1990 and 2021. In 1990, the peak incidence of NRCAVD occurred in the 60–64 age group for male individuals and the 65–69 age group for female individuals, showing a delay of 5 years compared to male individuals. By 2021, the highest incidence shifted to the 65–69 age group for both sexes. Regarding prevalence, the peak number of cases occurred in male individuals in the 65–69 age group and female individuals in the 70–74 age group in both 1990 and 2021. Both the ASRs of incidence and prevalence increased with age. For mortality, the peak number of cases was observed in male individuals in the 70–74 age group and female individuals in the 75–79 age group in both 1990 and 2021. The ASMR also increased with age across all female age groups and in male individuals up to the 94-year-old age group in 1990 and 2021. However, beyond the 95-year-old age group, there was a noticeable downward trend in male individuals. In terms of DALYs, the peak impact was observed in male individuals in the 65–69 age group and female individuals in the 70–74 age group in 1990. By 2021, the highest DALYs were recorded in the 70–74 age group for both sexes. Overall, these figures highlight an increasing burden of NRCAVD with age, particularly among older adults, and suggest a growing need for targeted healthcare interventions to address this trend.

### 3.5 The burden of NRCAVD in China and worldwide from 1990 to 2021

**Figure 6:**
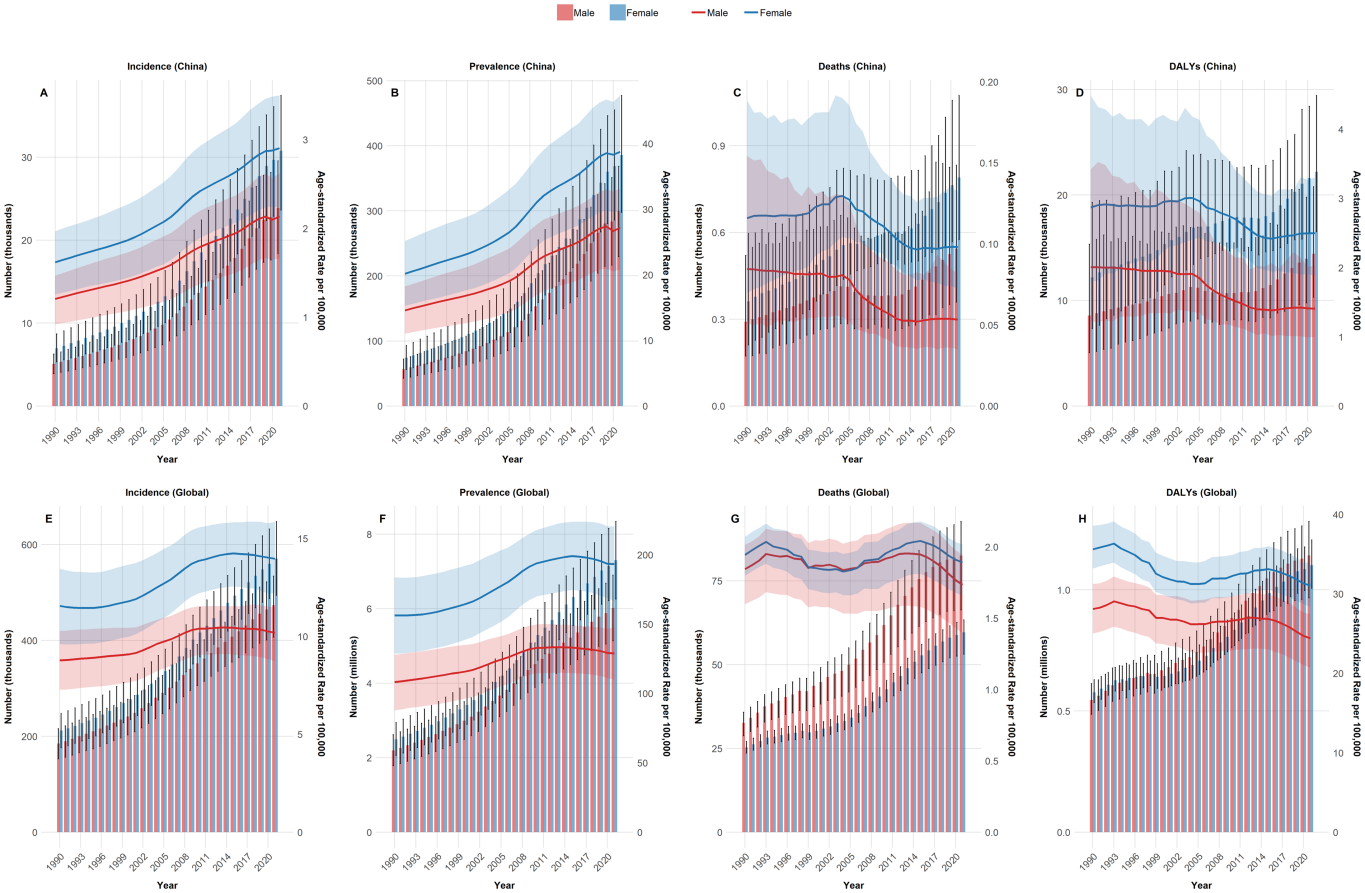
Comparison of all-age cases and the ASIR, ASPR, ASMR, and ASDALYs in male and female individuals in China and globally from 1990 to 2021. **(A)** Incidence in China; **(B)** Prevalence in China; **(C)** Deaths in China; **(D)** DALYs in China; **(E)** Global incidence; **(F)** Global Prevalence; **(G)** Global deaths; **(H)** Global DALYs. ASIR, age-standardized incidence rate; ASPR, age-standardized prevalence rate; ASMR, age-standardized mortality rate; ASDALYs, age-standardized DALYs rate; DALYs, disability-adjusted life years.

Figure 6 displays a comparison of the disease burden and its corresponding ASRs of NRCAVD in male and female individuals across all-age cases in China from 1990 to 2021. Over this period, the cases of incidence, prevalence, mortality, and DALYs exhibited a notable upward trend. Favorably, both the ASMR and ASDALYs showed a downward trend. It should be noted that, regardless of whether examining raw numbers or ASRs for these four metrics, male individuals consistently experienced higher rates than female individuals throughout the entire study period from 1990 to 2021.

As the four key metrics of NRCAVD (incidence, prevalence, mortality, and DALYs) in cases and ASIR, ASPR consistently increased globally from 1990 to 2021 in both sexes, it is clear that the international trend closely mirrors the pattern previously observed in China. Generally, male individuals exhibited higher values than female individuals in the four metrics, both in cases and their corresponding ASRs. However, globally, an intriguing contrast emerged: for mortality and DALY cases, female individuals had higher values than male individuals. These patterns were reversed in China. In addition, the ASDALYs demonstrated a downward trend. Unfortunately, the ASMR did not show a significant decrease over the period, displaying fluctuations instead (Figure 6).

### 3.6 Projection of the burden of NRCAVD until 2050 in China

**Figure 7:**
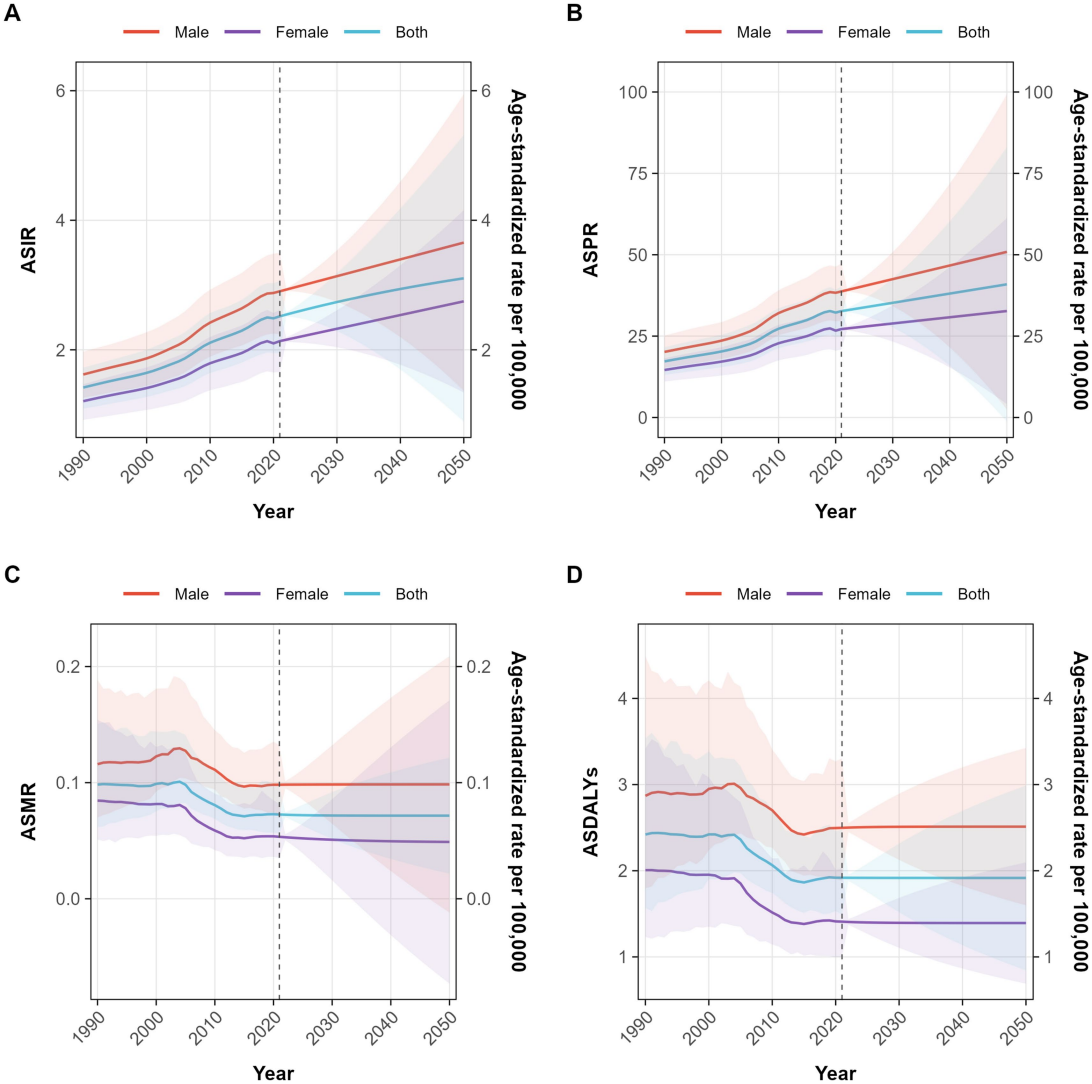
Projection of the ASIR, ASPR, ASMR, and ASDALYs of NRCAVD until 2050 in China. **(A)** ASIR, **(B)** ASPR, **(C)** ASMR, **(D)** ASDALYs. Abbreviations: ASIR, age-standardized incidence rate; ASPR, age-standardized prevalence rate; ASMR, age-standardized mortality rate; ASDALYs, age-standardized DALYs rate.

To obtain an overview of the epidemiological trends of NRCAVD in China over the next three decades, we employed Bayesian age-period-cohort models to project the number of cases and ASRs for incidence, prevalence, mortality, and DALYs. The projections indicated that by 2050, the cases of incidence will rise to 68,814 (95% CI: 60,435–77,193), and prevalence will increase to 905,286 (95% CI: 772,918–1,037,654). Fortunately, both the number of deaths and DALYs are expected to decrease, reaching 724 (95% CI: 477–970) and 18,892 (95% CI: 13,994–23,790), respectively. In addition, the ASIR and ASPR are projected to increase to 6.08 (95% CI: 5.3–6.82) per 100,000 and 79.98 (95% CI: 68.29–91.68) per 100,000 by 2050 (Figure 5). On a positive note, the ASMR and ASDALYs are anticipated to decrease to 0.06 (95% CI: 0.04–0.09) per 100,000 and 1.67 (95% CI: 1.23–2.10) per 100,000, respectively, by 2050. However, an unfortunate trend is that male individuals are projected to consistently experience higher rates than female individuals across all four key metrics until 2050 (Figure 7).

## 4 Discussion

In the study, we comprehensively evaluated the incidence, prevalence, mortality, and DALYs of NRCAVD in China and worldwide over the last 32 years based on the latest GBD 2021 database. We examined the variations in the disease burden of NRCAVD in China and globally. The results showed that the ASIR and ASPR of NRCAVD, both in China and globally, showed a significant increasing trend from 1990 to 2021, with rates increasing with age. In contrast, the ASMR and ASDALYs in China, as well as ASDALYs globally, showed a decreasing trend. However, the global ASMR trend exhibited fluctuations but showed no significant decrease, and the results were in line with prior reports (13, 14). Similar trajectory trends of the ASIR, ASPR, ASMR, and ASDALYs in China are expected to persist until 2050.

Non-rheumatic heart valve disease exhibits a strong age-related pattern, with prevalence primarily concentrated in individuals aged 60 and above. Due to population growth and aging, the prevalence and incidence of NRCAVD are expected to have a sharp increasing trend over the coming decades (15). Over the last three decades, the prevalence rate of NRCAVD increased more than 1.5-fold from 1990 to 2019, with approximately 9.40 million individuals with NRCAVD globally in 2019. The number of incidence cases increased more than 3.5-fold from 130,822 in 1990 to 589,638 in 2019 globally (13). A recent study showed that the number of prevalent cases reached 13,551,699, and the rate of prevalence was 156.6 per 100,000. The number of deaths reached 146,199, and the rate of death was 1.8 per 100,000. The DALY rate reached 26.8 per 100,000 globally in 2022 (16). Such a remarkably growing rate and cases significantly aggravate the medical burden of NRCAVD in older people, demanding our attention.

It is well established that aging is a driving factor for NRCAVD (17, 18). According to previous research, the greatest disease burden of NRCAVD occurs in the 70 + age group (13). We also found that the prevalence of NRCAVD remarkably increases with advancing age. The overwhelming majority of cases were concentrated in individuals over the age of 60, and the highest ASIR and ASPR of NRCAVD were observed in the 95 + age group. Prior research has shown obvious differences in the burden of NRCAVD among male and female individuals (42). In our study, we also found that male individuals had a higher ASIR and ASPR than female individuals, especially before the age of 75. The peak number of incidences in female individuals occurred 5 years later than in male individuals in 1990. This delay may be attributed to the fact that male individuals have more atherosclerotic risk factors, such as smoking, alcohol consumption, and more social pressure (19–21). In contrast, the protective effect of estrogen in female individuals may delay the onset of arteriosclerosis compared to male individuals (22–24). In contrast, the peak number of incidences was observed in the 65–69 age group for both male and female individuals in 2021. The peak age of incidence shifted to an older age in 2021 compared to 1990, a change that may be attributable to population aging and advances in healthcare. The ASMR increased with age in both male and female individuals up to 94 years old but showed a decreasing trend in male individuals after the age of 95 in China. A possible explanation for this turning point in the relationship between ASMR and age may be that NRCAVD and concurrent diseases with shared risk factors lead to early death in male individuals before the age of 95.

In the past 20 years, with the continuous accumulation of surgical experience and ongoing innovations in surgical equipment, TAVR surgery has advanced significantly. Its indications have gradually expanded from older patients with high surgical risk due to aortic stenosis to include more younger patients with lower surgical risk, as well as those with aortic regurgitation, a small valve ring, and aortic dilatation and even those requiring valve-in-valve TAVR (25–28). As a result, more and more patients are benefiting from it. The ASMR and ASDALYs of NRCAVD in China showed a downward trend from 2004 to 2021, indicating a favorable trend compared to the ASIR and ASPR of NRCAVD. This improvement may be attributed to profound advances in TAVR, especially after the world’s first successful TAVR procedure in 2002. Unfortunately, the global ASMR was relatively stable, not showing a significant decreasing trend in the last 30 years—especially among female individuals, whose number of deaths from NRCAVD was higher than that of male individuals. This result serves as a warning that more countries should pay more attention to older female individuals. Health authorities and policymakers in these areas need to increase resource allocation, improve healthcare services, and control variable risk factors to reduce the burden of NRCAVD. More than 20 years ago, the only treatment for patients with severe calcific aortic valve disease was surgical intervention, as no medical therapy options could alter the natural history of degenerative calcific aortic valve disease. Fortunately, the invention of TAVR has changed this dilemma. Its safety and efficacy have been confirmed through several randomized controlled studies (29–32). It was endorsed as a first-line therapy for patients with severe calcific aortic valve disease by both the ACC/AHA (2020) and ESC/EACTS (2021) guidelines. Notably, its indications have recently been expanded to include patients with pure native aortic regurgitation and younger populations, as outlined in the latest ESC/EACTS Valvular Heart Disease Management Guidelines updated 1 month ago (8, 33, 34). Considering the rapid advances achieved in TAVR, it may play an important role in reducing the burden of NRCAVD. It is necessary for these regions to introduce and popularize TAVR therapy in the future (35).

The divergent trends in the NRCAVD burden between China and the global average primarily reflect differences in socioeconomic development and public health policies. China’s rising prevalence (ASPR) aligns with its rapid urbanization and shifts toward Westernized diets and sedentary lifestyles, while the recent decline in mortality (ASMR) highlights improved healthcare access and advanced treatments (36). In contrast, the stable global ASMR and declining ASPR after 2015 likely result from effective large-scale interventions in many countries, such as sodium reduction programs and widespread statin use (37, 38). However, China’s increasing disability burden (ASDALYs) remains a concern, indicating growing morbidity despite improved survival.

This comparative analysis underscores China’s distinct NRCAVD trajectory, which is characterized by a rising absolute burden alongside a declining ASMR—a pattern that contrasts with global trends. This divergence reflects not only demographic pressures but also China’s effective mitigation of age-adjusted risks through public health measures (39, 40). The pronounced male-specific mortality disparity further highlights the need for sex-tailored interventions. These findings emphasize the importance of adapting global strategies to China’s specific context, while China’s experience may offer valuable insights for other nations undergoing similar epidemiological transitions.

The Bayesian age-period-cohort model analysis showed that the remarkably increasing trend in the incidence and prevalence of NRCAVD in China will continue until 2050. As the largest developing country, with continuous population growth and aging, the Chinese government needs ongoing resource investment, improved healthcare access, and more precise, targeted strategies to adapt to demographic structural changes for NRCAVD prevention and control, thereby reducing the disease burden of NRCAVD.

There are several limitations in the present study. First, partial missing data in the GBD database may adversely affect the accuracy of estimates, and the varying sources and quality of data across different countries in the GBD database may also introduce potential bias. Second, transthoracic echocardiography is less sensitive than cardiac computed tomography in detecting NRCAVD, yet NRCAVD was diagnosed using transthoracic echocardiography in most cases (41). Variations in diagnostic methods may lead to differences in the reported incidence and prevalence of NRCAVD. In addition, differences in diagnostic expertise and the quality of ultrasonic equipment among healthcare providers may also lead to differences in outcomes. Third, our prediction of the NRCAVD burden is limited by its reliance on past data and the sociodemographic index, as well as the core assumption that future risk factor trends will remain unchanged, which may be invalidated by real-world changes in factors such as policies, environments, and lifestyle. Fourth, the use of ICD-10 codes without distinguishing between different subtypes means that aortic stenosis and aortic regurgitation were combined in the GBD 2021 database. Detailed subtyping of NRCAVD is needed in the future to identify the burden of NRCAVD.

## Data Availability

The original contributions presented in the study are included in the article/supplementary material, further inquiries can be directed to the corresponding author/s.

## Footnotes

^1^ https://vizhub.healthdata.org/gbd-results

^2^ https://www.naturalearthdata.com
